# Implementation of Hospital Practices Supportive of Breastfeeding in the Context of COVID-19 — United States, July 15–August 20, 2020

**DOI:** 10.15585/mmwr.mm6947a3

**Published:** 2020-11-27

**Authors:** Cria G. Perrine, Katelyn V. Chiang, Erica H. Anstey, Daurice A. Grossniklaus, Ellen O. Boundy, Erin K. Sauber-Schatz, Jennifer M. Nelson

**Affiliations:** ^1^Division of Nutrition, Physical Activity, and Obesity, National Center for Chronic Disease Prevention and Health Promotion, CDC; ^2^Oak Ridge Institute for Science and Education, Oak Ridge, Tennessee ^3^CDC COVID-19 Response Team.

Breastfeeding has health benefits for both infants and mothers and is recommended by numerous health and medical organizations*^,†^ (*1*). The birth hospitalization is a critical period for establishing breastfeeding; however, some hospital practices, particularly related to mother-newborn contact, have given rise to concern about the potential for mother-to-newborn transmission of SARS-CoV-2, the virus that causes coronavirus disease 2019 (COVID-19) (*2*). CDC conducted a COVID-19 survey (July 15–August 20, 2020) among 1,344 hospitals that completed the 2018 Maternity Practices in Infant Nutrition and Care (mPINC) survey to assess current practices and breastfeeding support while in the hospital. Among mothers with suspected or confirmed COVID-19, 14.0% of hospitals discouraged and 6.5% prohibited skin-to-skin care; 37.8% discouraged and 5.3% prohibited rooming-in; 20.1% discouraged direct breastfeeding but allowed it if the mother chose; and 12.7% did not support direct breastfeeding, but encouraged feeding of expressed breast milk. In response to the pandemic, 17.9% of hospitals reported reduced in-person lactation support, and 72.9% reported discharging mothers and their newborns <48 hours after birth. Some of the infection prevention and control (IPC) practices that hospitals were implementing conflicted with evidence-based care to support breastfeeding. Mothers who are separated from their newborn or not feeding directly at the breast might need additional postdischarge breastfeeding support. In addition, the American Academy of Pediatrics (AAP) recommends that newborns discharged before 48 hours receive prompt follow-up with a pediatric health care provider.

Peripartum practices, including immediate maternal-newborn skin-to-skin contact, enabling mothers and newborns to room-in together, and teaching mothers to breastfeed (e.g., demonstrating good positioning and attachment and supporting mothers to express breast milk if they are temporarily separated from their newborns), are recommended worldwide for implementation in birth hospitals (*3*,*4*). These practices have a positive impact on both short- and long-term breastfeeding outcomes, which in turn benefit maternal and child health (*5*,*6*). Guidance on care during the birth hospitalization has evolved with the pandemic and at times has varied across public health and professional medical organizations.

CDC’s mPINC survey is a census of all birth hospitals in the United States and its territories.^§^ The 2,039 hospitals that completed the 2018 mPINC survey (70% response) were sent an e-mail link to a 13-item COVID-19 survey, conducted using Research Electronic Data Capture (REDCap) (version 10.0.08; Vanderbilt University). The survey was open during July 15–August 20, 2020 and asked about current hospital practices. Maternity services had been closed at 22 hospitals, and 130 e-mailed surveys were undeliverable (i.e., e-mails bounced back). Overall, 1,344 hospitals completed the survey (66.6% overall response rate; 71.2% among delivered surveys). Hospitals were asked about their actual or planned approach to managing maternity patients with suspected or confirmed COVID-19 (as defined by the hospital), and the approximate number of these patients they had cared for. Descriptive analyses were conducted using SAS software (version 9.4; SAS Institute). This activity was reviewed by CDC and was conducted consistent with applicable federal law and CDC policy.^¶^ Data on annual births and hospital type were obtained from the 2018 mPINC survey.

Among 1,343 hospitals with available information, 724 (53.9%) had cared for one to 19 newborns whose mothers had confirmed COVID-19; 152 (11.4%) had cared for 20 or more, and 457 (34.0%) had not cared for any (Table 1). Approximately one half of the hospitals reported fewer than 1,000 annual births, 42.0% reported 1,000–4,999, and 2.8% reported 5,000 or more. The majority (78.8%) of hospitals were nonprofit.

**TABLE 1:** Characteristics of hospitals participating in CDC’s Maternity Practices in Infant Nutrition and Care (mPINC) COVID-19 survey — U.S. states and territories, July 15–August 20, 2020

Characteristic	No. (%)
**Total responding hospitals**	1,344 (100.0)
No. of infants born to mothers with confirmed COVID-19 since the start of the pandemic*	
0	457 (34.0)
1–19	724 (53.9)
20–59	111 (8.3)
60–99	24 (1.8)
≥100	17 (1.3)
Do not know	10 (0.7)
**Hospital type** **^†^**	
Nonprofit	1,059 (78.8)
Private	221 (16.4)
Government/Military	64 (4.8)
**Hospital annual births** **^†^**	
<500	438 (32.6)
500–999	305 (22.7)
1,000–1,999	285 (21.2)
2,000–4,999	279 (20.8)
≥5,000	37 (2.8)

**Abbreviation:** COVID-19 = coronavirus disease 2019.

* Because of a missing response, the sample size for this question is 1,343. Hospitals were asked to estimate the approximate number of infants born to mothers with confirmed COVID-19; no definition of confirmed COVID-19 was provided.

^†^ Hospital type and annual births were reported by hospitals in the 2018 mPINC survey.

Among 1,344 birth hospitals, 1,211 (90.2%) reported having enough COVID-19 tests, and 864 (64.3%) were performing universal COVID-19 testing of women admitted to labor and delivery (Table 2). Few hospitals (4.8%) reported separating all mothers and newborns until the mother received a negative test result, and 28.6% separated newborns and mothers if the mother was symptomatic or had a known exposure until test results were obtained; 24.2% separated mothers and newborns only if the mother received a positive test result.

**TABLE 2:** Hospital maternity care practices and breastfeeding support in the context of the COVID-19 pandemic — U.S. states and territories, July 15–August 20, 2020

Hospital maternity care practices (no. with available information)	No. (%)
Universal COVID-19 testing among women admitted to labor and delivery (1,344)	**864 (64.3)**
Adequate COVID-19 tests available for women admitted to labor and delivery (1,343)	**1,211 (90.2)**
**Is hospital separating mothers and newborns until the mother receives a negative COVID-19 test? (1,322)**	
Yes, all newborns are separated until mother receives a negative result	64 (4.8)
No, newborns are only separated from mothers with symptoms or known exposure while awaiting results	378 (28.6)
No, all mothers and newborns remain together until the mother receives a positive result	320 (24.2)
No, all mothers and newborns are kept together regardless of symptoms, known exposure, or test results	560 (42.4)
**Skin-to-skin care in the first hour after birth of a healthy newborn whose mother has suspected/confirmed COVID-19 (1,335)**	
Encouraged	178 (13.3)
Determined case-by-case as shared decision with the mother	883 (66.1)
Discouraged	187 (14.0)
Prohibited	87 (6.5)
**Rooming-in for newborns of mothers with suspected/confirmed COVID-19 (1,334)**	
Encouraged; no precautions required	34 (2.6)
Encouraged with precautions to maintain distance	726 (54.4)
Discouraged, but allowed if mother’s preference	504 (37.8)
Prohibited; newborn was cared for in a room separate from mother	70 (5.3)
**Breastfeeding for mothers with suspected/confirmed COVID-19 (1,334)**	
Direct breastfeeding encouraged with precautions (e.g., mask, handwashing)	893 (66.9)
Direct breastfeeding discouraged but allowed with precautions if mother chooses	268 (20.1)
Direct breastfeeding not supported, but mothers encouraged to express breast milk for feeding by a healthy caregiver	170 (12.7)
Formula feeding recommended	3 (0.2)
**Mothers with suspected/confirmed COVID-19 who are not breastfeeding are supported to start expressing breast milk (1,316)**	
Within 1 hr of birth	438 (33.3)
1–3 hrs after birth	645 (49.0)
4–6 hrs after birth	195 (14.8)
Timing is not a consideration	34 (2.6)
Mothers are discouraged from expressing breast milk	4 (0.3)
**Because of the COVID-19 pandemic, direct lactation support has decreased (1,339)**	239 (17.9)
**Because of the COVID-19 pandemic, hospital is discharging mothers and newborns <48 hrs after birth (1,337)**	975 (72.9)
Postdischarge breastfeeding support currently offered by the hospital* (1,344)	
In-person breastfeeding support consultations	802 (59.7)
Virtual breastfeeding consultations	655 (48.7)
Information on how to access a breast pump	1,047 (77.9)
Renting or lending hospital-grade breast pumps	469 (34.9)
**Hospital exclusive breastfeeding rate since start of the pandemic (1,341)**	
Increased	152 (11.3)
Decreased	164 (12.2)
Stayed about the same	924 (68.9)
Don’t know	101 (7.5)

**Abbreviation:** COVID-19 = coronavirus disease 2019.

* Hospital could indicate all types of discharge support that applied.

Overall, 178 (13.3%) hospitals encouraged skin-to-skin contact between mothers with suspected or confirmed COVID-19 and their newborns immediately after birth, and 883 (66.1%) decided this on a case-by-case basis; 187 (14.0%) hospitals discouraged, and 87 (6.5%) prohibited skin-to-skin contact between mothers with suspected or confirmed COVID-19 and their newborns. Approximately one half of hospitals (726; 54.4%) encouraged rooming-in for mothers with suspected or confirmed COVID-19, with precautions to maintain distance, whereas 504 (37.8%) discouraged and 70 (5.3%) prohibited rooming-in. Approximately two thirds of hospitals supported direct breastfeeding with precautions (e.g., mask use and handwashing) for mothers with suspected or confirmed COVID-19 (893; 66.9%), whereas 268 (20.1%) discouraged direct breastfeeding but would allow it according to mother’s choice, and 170 (12.7%) did not support direct breastfeeding but encouraged expressed breast milk feeding by a healthy caregiver. When mothers with suspected or confirmed COVID-19 were not breastfeeding directly, 438 (33.3%) hospitals reported supporting expression of breast milk within 1 hour of birth, and 645 (49.0%) within 1–3 hours.

Because of the COVID-19 pandemic, 239 (17.9%) hospitals reported decreased access to in-person lactation support and 72.9% discharged mothers and newborns <48 hours after birth. After discharge, 802 (59.7%) and 655 (48.7%) hospitals offered in-person and virtual breastfeeding consultations, respectively. Since the start of the pandemic, 924 hospitals (68.9%) reported that their exclusive breastfeeding rates during hospitalization had stayed about the same, and similar percentages reported increases (11.3%), compared with decreases (12.2%).

## Discussion

During a 5-week period (July 15–August 20) when many areas of the country were experiencing substantial community transmission of SARS-CoV-2, hospitals were implementing a variety of practices intended to balance evidence-based maternity care with COVID-19-related IPC. Hospital practices are likely evolving along with the pandemic (*7*), potentially driven by multiple factors, including level of community transmission, guidance from public health and medical professional organizations, and a hospital’s own experience in preparation for or caring for pregnant women and newborns with COVID-19.

Various organizations have promulgated COVID-19 guidance on care for pregnant women and newborns, which at times has been conflicting. The World Health Organization recommends that mothers with COVID-19 be able to practice skin-to-skin care, rooming-in, and direct breastfeeding while wearing a mask, unless they are too ill to do so**; similar guidance is supported by the American Academy of Family Physicians,^††^ and the American College of Obstetricians and Gynecologists^§§^ promotes shared decision-making with the mother and the health care team. On the other hand, when this survey was launched, CDC and AAP recommended temporary separation of newborns from mothers with suspected or confirmed COVID-19 (*8*). During data collection for this survey, CDC (August 3^¶¶^) and AAP (July 22***) updated their guidance, supporting maternal autonomy in decision-making. Changes in guidance reflect evolving knowledge about the virus and its potential impact on newborns. To date, there has been no definitive evidence of transmission of SARS-CoV-2 through breast milk. In addition, there have been reports of secretory immunoglobulin A against SARS-CoV-2 in breast milk samples of women with COVID-19, suggesting that breastfeeding might be particularly important for newborns of mothers with COVID-19 (*9*).

One third of hospitals in this study reported not having cared for any neonates born to mothers with COVID-19; however, others had extensive experience, including 17 hospitals reporting caring for at least 100 of these newborns. Follow-up of mothers with COVID-19 delivering at three large New York birth hospitals found reduced breastfeeding rates both in the hospital and after returning home among mothers who had been separated from their newborns (*7*). After identification of this finding, and the observed stress among mothers and newborns as a result of separation, the hospital system revised its policy and began to allow asymptomatic mothers with laboratory-confirmed COVID-19 to room-in and breastfeed.

Nearly one in five hospitals in this study reported that in-person lactation support had decreased during the pandemic. Approximately 60% of hospitals in this study reported offering in-person breastfeeding consultations postdischarge, compared with 69% of hospitals reporting offering this in the 2018 mPINC survey (CDC, unpublished data, 2020). Lactation specialists working in health care settings should follow recommended IPC measures for those settings.^†††^ Nearly one half of hospitals reported offering virtual breastfeeding consultations; however, no data are available on this practice before the pandemic. Notably, these changes in lactation support affect newborns broadly, not just those born to mothers with COVID-19.

Approximately equal numbers of hospitals reported that their exclusive breastfeeding rates had increased and decreased; the majority reported the rate had stayed approximately the same. The reasons for these changes are unknown. However, the pandemic could contribute to reduced breastfeeding as a result of maternal/newborn separation and reductions in lactation support. On the other hand, the visitor restriction policies implemented by many hospitals could potentially provide more opportunities for breastfeeding and for a mother to learn her newborn’s feeding cues.^§§§^ Longer-term monitoring of exclusive breastfeeding rates at hospital discharge will be important to assess as one measure of the impact of the pandemic on infant health.

Approximately three fourths of hospitals reported discharging mothers and their newborns <48 hours after birth because of the pandemic. No standard length of stay for the birth hospitalization exists, but the Newborn Mothers’ Health and Protection Act of 1996 prohibits the restriction of benefits to <48 hours for a vaginal delivery or <96 hours for a cesarean section.^¶¶¶^ AAP describes discharge <48 hours after delivery as a “shortened hospital stay” and notes that although it can be accommodated for healthy term newborns, it is not appropriate for all mothers and infants (*10*). AAP also recommends that all newborns discharged <48 hours after birth be evaluated by a pediatric health care provider within 48 hours of discharge (*10*). This visit, in part, assesses effective feeding, which might be more critical during the pandemic when breastfeeding mothers might be receiving reduced hospital lactation support.

The findings in this report are subject to at least three limitations. First, although mPINC is a census of all birth hospitals, this survey was only sent to the hospitals that completed the 2018 mPINC survey. Second, hospitals used their own definitions for suspected and confirmed COVID-19, and those definitions might have been different. Finally, data captured in this survey represent a single point in time. Policies and practices likely will continue to change as the pandemic evolves.

Women with suspected or confirmed COVID-19 who are separated from their newborns and whose newborns are not feeding directly at the breast might need timely, professional, breastfeeding support.**** In addition, AAP advises that infants discharged <48 hours after delivery receive prompt follow-up with a pediatric health care provider to ensure optimal feeding.

**Summary**

**What is already known about this topic?**

Evidence-based hospital practices supporting breastfeeding have sometimes conflicted with COVID-19 infection prevention and control measures.

**What is added by this report?**

During summer 2020, hospitals implemented a variety of practices intended to balance evidence-based maternity care with infection prevention and control. Because of the pandemic, 17.9% of hospitals reported that in-person lactation support had decreased, and 72.9% reported discharging mothers and their babies <48 hours after birth.

**What are the implications for public health practice?**

Additional postdischarge breastfeeding support and newborn follow-up might be needed during the COVID-19 pandemic. Longer-term monitoring of exclusive breastfeeding rates at hospital discharge will be important for assessing this aspect of the pandemic on infant health.

## Footnotes

* https://www.cdc.gov/breastfeeding/about-breastfeeding/why-it-matters.html.

§ https://www.cdc.gov/breastfeeding/data/mpinc.

¶ 45 C.F.R. part 46, 21 C.F.R. part 56; 42 U.S.C. Sect. 241(d); 5 U.S.C. Sect. 552a; 44 U.S.C. Sect. 3501 et seq.

** https://www.who.int/news-room/commentaries/detail/breastfeeding-and-covid-19.

†† https://www.aafp.org/about/policies/all/breastfeeding-covid19.html.

§§ https://www.acog.org/en/Clinical/Clinical%20Guidance/Practice%20Advisory/Articles/2020/03/Novel%20Coronavirus%202019.

¶¶ https://www.cdc.gov/coronavirus/2019-ncov/hcp/caring-for-newborns.html.

*** https://services.aap.org/en/pages/2019-novel-coronavirus-covid-19-infections/clinical-guidance/faqs-management-of-infants-born-to-covid-19-mothers/.

††† https://www.cdc.gov/coronavirus/2019-ncov/hcp/infection-control-recommendations.html.

§§§ https://www.medscape.com/viewarticle/933964.

¶¶¶ https://www.cms.gov/CCIIO/Programs-and-Initiatives/Other-Insurance-Protections/nmhpa_factsheet.

**** https://www.cdc.gov/coronavirus/2019-ncov/hcp/care-for-breastfeeding-women.html.

## References

[1] American Academy of Pediatrics. Section on breastfeeding. Breastfeeding and the use of human milk. Pediatrics 2012;129:e827–41. 10.1542/peds.2011-3552 22371471

[2] Davanzo R, Merewood A, Manzoni P. Skin-to-skin contact at birth in the COVID-19 era: in need of help! Am J Perinatol 2020;37(Suppl 02):S1–4. 10.1055/s-0040-1714255 32772355

[3] World Health Organization. Implementation guidance: protecting, promoting and supporting breastfeeding in facilities providing maternity and newborn services—the revised Baby Friendly Hospital Initiative. Geneva, Switzerland: World Health Organization; 2018. https://www.who.int/nutrition/publications/infantfeeding/bfhi-implementation-2018.pdf

[4] US Department of Health and Human Services. The Surgeon General’s call to action to support breastfeeding. Washington, DC: US Department of Health and Human Services, Office of the Surgeon General; 2011. https://www.ncbi.nlm.nih.gov/books/NBK52682/

[5] Pérez-Escamilla R, Martinez JL, Segura-Pérez S. Impact of the Baby-friendly Hospital Initiative on breastfeeding and child health outcomes: a systematic review. Matern Child Nutr 2016;12:402–17 10.1111/mcn.12294 26924775 PMC6860129

[6] Feltner C, Weber RP, Stuebe AM, Grodensky CA, Orr C, Viswanathan M. Breastfeeding programs and policies, breastfeeding uptake, and maternal health outcomes in developed countries. Rockville, MD: Agency for Healthcare Research and Quality; 2018. https://effectivehealthcare.ahrq.gov/products/breastfeeding/research 30204377

[7] Popofsky S, Noor A, Leavens-Maurer J, Impact of maternal severe acute respiratory syndrome coronavirus 2 detection on breastfeeding due to infant separation at birth. J Pediatr 2020;226:64–70. 10.1016/j.jpeds.2020.08.004 32791077 PMC7416110

[8] Puopolo KM, Hudak ML, Kimberlin DW, Cummings J; Committee on Fetus and Newborn, Section on Neonatal Perinatal Medicine and Committee on Infectious Disease. Initial guidance: management of infants born to mothers with COVID-19. Itasca, IL: American Academy of Pediatrics; 2020. https://www.tn.gov/content/dam/tn/health/documents/cedep/novel-coronavirus/AAP_COVID-19-Initial-Newborn-Guidance.pdf

[9] Fox A, Marino J, Amanat F, Robust and specific secretory IgA against SARS-CoV-2 detected in human milk. iScience 2020;23:101735. 10.1016/j.isci.2020.101735 33134887 PMC7586930

[10] Benitz WE; Committee on Fetus and Newborn, American Academy of Pediatrics. Hospital stay for healthy term newborn infants. Pediatrics 2015;135:948–53. 10.1542/peds.2015-0699 25917993

